# Improving safety of the continual reassessment method via a modified allocation rule

**DOI:** 10.1002/sim.8450

**Published:** 2019-12-20

**Authors:** Pavel Mozgunov, Thomas Jaki

**Affiliations:** ^1^ Department of Mathematics and Statistics Lancaster University Lancaster UK

**Keywords:** allocation rule, continual reassessment method, loss function, phase I clinical trial, restricted parameter space

## Abstract

This article proposes a novel criterion for the allocation of patients in phase I dose‐escalation clinical trials, aiming to find the maximum tolerated dose (MTD). Conventionally, using a model‐based approach, the next patient is allocated to the dose with the toxicity estimate closest (in terms of the absolute or squared distance) to the maximum acceptable toxicity. This approach, however, ignores the uncertainty in point estimates and ethical concerns of assigning a lot of patients to overly toxic doses. In fact, balancing the trade‐off between how accurately the MTD can be estimated and how many patients would experience adverse events is one of the primary challenges in phase I studies. Motivated by recent discussions in the theory of estimation in restricted parameter spaces, we propose a criterion that allows to balance these explicitly. The criterion requires a specification of one additional parameter only that has a simple and intuitive interpretation. We incorporate the proposed criterion into the one‐parameter Bayesian continual reassessment method and show, using simulations, that it can result in similar accuracy on average as the original design, but with fewer toxic responses on average. A comparison with other model‐based dose‐escalation designs, such as escalation with overdose control and its modifications, demonstrates that the proposed design can result in either the same mean accuracy as alternatives but fewer toxic responses or in a higher mean accuracy but the same number of toxic responses. Therefore, the proposed design can provide a better trade‐off between the accuracy and the number of patients experiencing adverse events, making the design a more ethical alternative over some of the existing methods for phase I trials.

## 1 INTRODUCTION

Consider a phase I clinical trial with two doses (*d*_1_, *d*_2_) and a binary endpoint, dose‐limiting toxicity (DLT) or no DLT, to stress the importance of an allocation criterion. The goal of the trial is to find the maximum tolerated dose (MTD), which has a probability of a DLT closest to the prespecified target, say γ=0.30. Assume that 10 patients were assigned to each dose and two and four toxicities are observed, respectively. Then, a typical question in a sequential trial is “which dose should be administered to the next patient.” A conventional criterion for many model‐based dose‐escalation designs^1^, ^2^ is to assign the next patient to dose *d*_*i*_ corresponding to the point estimate ${\hat{p}}_{i}$ closest to γ in terms of the absolute or, equivalently, the squared distance

(1) $${\left({\hat{p}}_{i}-\gamma \right)}^{2}.$$

Assume that in the example above, the probabilities *p*_1_ and *p*_2_ are considered as random variables with beta distributions ℬ(2,8) and ℬ(4,6) and one uses the mean as the point estimate: ${\hat{p}}_{1}=0.2$ and ${\hat{p}}_{2}=0.4$. Following criterion (1), the next patient can be allocated to either dose as both estimates are equally close to the target. At the same time, one can argue that these doses are not “equal” for at least two reasons. On the one hand, criterion (1) ignores the randomness of the estimates. Indeed, the probability of being within 5% of γ is larger for *p*_2_

(2) $$P(p_{2}\in (0.25,0.35))>P(p_{1}\in (0.25,0.35)).$$

The larger variance of *p*_2_ favors the decision to allocate the next patient to *d*_2_. On the other hand, allocating a patient to a dose with an estimated toxicity probability of 0.4 might be considered unethical as it exposes a patient to unacceptably high toxicity. Clearly, the squared distance criterion (1) fails to account for the uncertainty in the estimates and allows patients to be allocated to overly toxic doses. One of the main challenges in phase I dose‐escalation trials is, however, to balance the trade‐off between accurately estimating the MTD and minimizing the number of adverse events in the study.^3^

The question of safety was first addressed using the escalation with overdose control (EWOC) design.^4^ The EWOC uses the criterion

(3) $$E(\alpha {\left(\gamma -p_{i}\right)}^{+}+(1-\alpha ){\left(p_{i}-\gamma \right)}^{+}),$$

for patients allocations,^5^ where ${\left(x\right)}^{+}=\max(0,x)$ and α is a parameter of asymmetry. The criterion (3) imposes that the allocation to a more toxic dose should have a more severe penalty than the allocation to a less toxic dose. The EWOC design has been shown to result in a low average number of DLTs. However, it also leads to an underestimation of the MTD in many realistic scenarios, and some modifications were recently proposed.^6^, ^7^ The main objective of these modifications is to achieve a better trade‐off between accuracy and a number of adverse events. Generally, it was proposed to change the parameter α as the trial progresses to increase the accuracy while not compromising safety. These approaches, however, use nontrivial functions for the time (or/and toxicity)‐ dependent parameter α, which might require substantial calibration.

Another design aimed at tackling the accuracy‐toxicity trade‐off is the Bayesian logistic regression model (BLRM).^8^ To address the first concern, it is proposed to use the whole distribution of the DLT probability, while the safety aspect is addressed using a penalty to overly toxic doses. The allocation is determined by a loss function computed for each dose. Although this approach has been proven to be useful in practice, it also requires specification of several parameters that need to be calibrated and on which the operating characteristics of the design hinges.

Interestingly, both the BLRM and modifications to the EWOC are building upon a two‐parameter logistic model, while the problem of addressing the accuracy‐toxicity trade‐off for a one‐parameter continual reassessment method (CRM) design^1^ through the allocation criterion has received little attention in the dose‐finding literature. Commonly, ad hoc practical solutions are to either (i) assign patients to the dose closest but below the estimated MTD dose or (ii) to assign patients to a lower target probability (eg, 0.25 when seeking 0.33). It is clear that both of these approaches will result in underestimation of the MTD. Moreover, such approaches are inflexible in terms of the accuracy‐toxicity trade‐off that can be achieved. Consequently, a more statistically solid approach is required.

In this work, we propose a new criterion for the allocation of patients in dose‐escalation trials, which is based on recent developments in the estimation of the restricted parameter space^9^ and provides a statistical basis for the inclusion of safety concerns into the decision making. The proposed criterion controls the trade‐off between the uncertainty in estimates and the conservatism of an investigator (in terms of the mean number of toxic responses) and requires only one additional parameter, which has a simple and intuitive interpretation, to be specified. As it is generally agreed that model‐based phase I designs lead to improved operating characteristics than rule‐based alternatives,^10^ we incorporate the proposed criterion into the Bayesian CRM^1^ that uses a one‐parameter power model and compare its operating characteristics with the traditional one‐parameter power model CRM design, the EWOC design and its recent modifications, and the BLRM design. We show the proposed design for particular levels of the trade‐off parameter can achieve either (i) the same average accuracy as many of currently employed methods but with fewer patients experiencing adverse events or (ii) obtain higher average accuracy than EWOC but the same number of adverse events. We show that the proposed design offers a more ethical alternative to these methods as it offers a better accuracy‐toxicity trade‐off.

The remainder of the article proceeds as follows. The new criterion and its properties are studied in Section 2. The application of the novel criterion in the context of an actual clinical trial is considered in Section 3. A simulation comparison with the traditional CRM is given in Section 4. A comparison with a range of alternative approaches is given in Section 5. Section 6 concludes with a discussion.

## 2 METHODS

### 2.1 Criterion

Consider a phase I clinical trial with binary DLT outcomes and *m* doses *d*_1_,…,*d*_*m*_. The main estimation objective in a phase I trial is the probability of DLT, *p*_*i*_∈(0,1), if dose *d*_*i*_ was given to a patient. Once estimates of *p*_*i*_ are obtained, an investigator selects the MTD as the dose associated with the toxicity probability closest to γ∈(0,1). Let us consider the criterion (1) for some fixed dose *d* with associated probability *p*. It has been argued for a long time in various areas of statistics^11^, ^12^ that the squared distance criterion (1) might not be a reliable measure of distance between objects defined on restricted parameter spaces. This argument is also valid in the considered phase I setting as both *p* and γ are defined on the restricted space—the unit interval. To tackle this problem, the objects on the unit interval could be linearized using the logit trasnformation. Indeed, the squared distance between such linearized objects was proposed as a distance between *p* and γ^11^

(4) $$\text{AD}(p,\gamma )=\sqrt{{\left(\log\frac{p}{1-p}-\log\frac{\gamma }{1-\gamma }\right)}^{2}},\;p,\gamma \in (0,1)$$

and is known as the Aitchison distance. Although the Aitchison distance was proven to be a useful tool in the compositional data analysis,^12^ it was recently noted that the Aitchison distance lacks some important properties such as convexity and a closed form solution for the corresponding minimizer.^9^ Moreover, one can argued that clinicians might encounter particular difficulties with interpreting the distance (4) in an actual trial. Instead, the convex unit‐interval‐symmetric divergence

(5) $$\delta (p,\gamma )=\frac{{\left(p-\gamma \right)}^{2}}{p(1-p)}.$$

was proposed.^9^ The symmetry of the divergence is defined in terms of the squared distance after the logit transformation. Specifically, it was found that for every choice of *p*_1_≤γ≤*p*_2_∈(0,1),

(6) $$\text{logit}(\gamma )-\text{logit}(p_{1})=\text{logit}(p_{2})-\text{logit}(\gamma ),$$

implies the equality of the proposed criteria δ(γ,*p*_1_)=δ(γ,*p*_2_). Therefore, it behaves similar to the logistic transformation while preserving convexity. Importantly, the divergence (5) reassembles a Wald‐type statistic consisting of the squared distance in the numerator and the variance of the probability of a binary event in the denominator. Thereby, the divergence (5) takes the uncertainty of the estimation object into account.

It was found that using this divergence as a loss function in several classic Bayesian problems with parameters defined on the unit interval can provide benefits in terms of the accuracy of the estimation.^9^ In this work, we propose to use a generalization of this divergence to govern dose selection in a dose‐finding study. Note that the measure (5) takes its minimum value, δ(·)=0, at *p*=γ. Due to the denominator, if *p*=0 or *p*=1, then δ(·)=∞, meaning that patients would be never allocated to doses corresponding to 0 or 1 DLT probabilities. Indeed, the property of assigning of infinite values to the extreme values “drives away” the selection from the bounds to the neighborhood of the interval of interest γ. 13, 14 Importantly, the criterion (5) also has an information‐theoretic justification as it maximizes the asymptotic information gain in the trial with a special interest in the neighborhood of the maximum acceptable toxicity.^15^

Applying the criterion to the illustration example above helps to address the uncertainty issue as

$$\delta ({\hat{p}}_{1}=0.2,\gamma =0.3)=1/16\;\text{and}\;\delta ({\hat{p}}_{2}=0.4,\gamma =0.3)=1/24.$$

This means that *d*_2_ should be selected for a next patient as follows from Inequality (2). Note that a single‐point estimate of the criterion (5) already summarizes the information about uncertainty in itself, which can provide a potential computational benefits.

The target toxicity γ is always less than 0.5 in phase I clinical trials. Consequently, if one would consider two‐point estimates that stand on the same squared distance (γ−θ)^2^ from the γ (for θ<γ), the criterion (5) favors a higher probability estimate due to the variance term in the denominator, which is maximized at the point *p*=0.5. Indeed, the same rate of terms *p* and (1−*p*) in the denominator implies that overly toxic and overly safe doses are equally penalized. As this, however, contradicts the ethical concerns of dose‐escalation trials, we propose a generalization of the measure, allowing for asymmetric penalization of doses below and above the target toxicity.

### 2.2 Asymmetry parameter

We generalize the criterion (5) to the case of asymmetric penalization by including the asymmetry parameter *a*:

(7) $$\delta (p,\gamma )=\frac{{\left(p-\gamma \right)}^{2}}{p^{a}{\left(1-p\right)}^{2-a}}.$$

**Figure 1:**
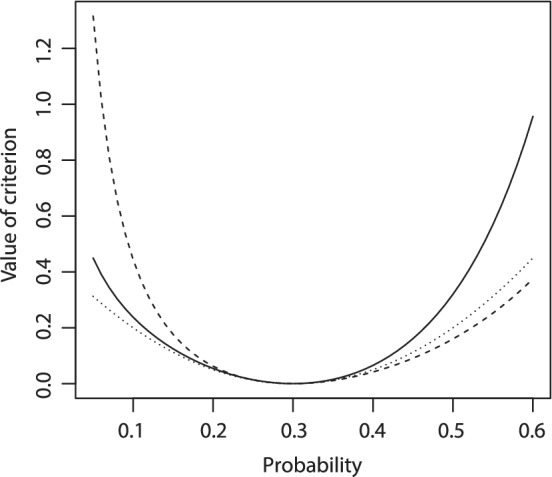
Squared distance criterion (dotted line) and CIBP criterion using the asymmetry parameter *a*=1 (dashed line), *a*=0.5 (solid line) for the target toxicity *γ*=0.3, and for different values *p*∈(0.05,0.6)

The parameter 0<*a*<2 corresponds to the penalization of overly toxic doses and 2−*a* to overly safe doses. The constant 2 is chosen to preserve the same rate of *p* in both nominator and denominator to guarantee that δ→0 when *p*→γ for all values of γ. Clearly, values 0<*a*<1 imply a more severe penalty for the allocation of patients to more toxic doses than to less toxic ones. Applying the proposed criterion with asymmetry parameter *a*=0.5, one can obtain that

$$\delta ({\hat{p}}_{1}=0.2,\gamma =0.3,a=0.5)<\delta ({\hat{p}}_{2}=0.4,\gamma =0.3,a=0.5),$$

which means that dose *d*_1_ would be selected due to the penalty on overly toxic doses. We will refer to the proposed criterion (7) as to the convex infinite bounds penalization (CIBP). An illustration of the squared distance criterion (1) and of the CIBP criterion (5) using *a*=1 and *a*=0.5 is given in Figure 1.

The CIBP criterion (for both *a*=1 and *a*=0.5) goes to infinity faster than the squared distance as the probability, *p*, approaches the lower bound. At the same time, for *a*=1, overly toxic doses are penalized less than by alternatives because corresponding values of the toxicity probability are located far from another boundary value 1. The asymmetric CIBP criterion with *a*=0.5 solves this issue and penalizes overly toxic doses more severely than both the squared distance and the symmetric CIBP. Note that all criteria behave similarly in the neighborhood of the target γ. Overall, one can see that the properties of the proposed criterion allow resolving the ethical concern by setting an appropriate value of the parameter *a*. Further guideline on the choice of *a* is given in the following section.

### 2.3 Choice of the asymmetry parameter

First, note that the denominator alone is maximized at the point *p*=*a*/2. Then, if $\hat{p}$ is an estimator of *p* (depending on the approach, for instance, MLE or the Bayesian optimal estimator), the “plug‐in” estimator of the CIBP criterion

(8) $$\delta (\hat{p},\gamma )=\frac{{\left(\hat{p}-\gamma \right)}^{2}}{{\hat{p}}^{a}{\left(1-\hat{p}\right)}^{2-a}},$$

using *a*=2γ leads to the same allocation as a plug‐in estimator of the squared distance (1). Then, values *a*<2γ imply a more conservative allocation of patients than an original design that uses the squared distance criterion.

Second, the asymmetry parameter *a* represents the trade‐off between the ethical and uncertainty concerns. Then, for a sensible choice of *a*, we use the following condition. Consider an interval (γ−θ,γ+θ). Assume that given two‐point toxicity probability estimates belonging to this interval and standing on the same squared distance from γ, one would like to select the lower toxicity estimate due to the safety concern. In other words, (γ−θ,γ+θ) is the interval in which the safety issue is prioritized. Similarly, given two estimates lying outside of the interval (γ−θ,γ+θ), but standing on the same squared distance, one would select that one which corresponds to a higher level of the uncertainty. Evidently, the estimates lying on the bounds of this interval should correspond to the same value of the CIBP criterion. Formally, solving

$$\frac{{\left((\gamma -\theta )-\gamma \right)}^{2}}{{\left(\gamma -\theta \right)}^{a}{\left(1-(\gamma -\theta )\right)}^{2-a}}=\frac{{\left((\gamma +\theta )-\gamma \right)}^{2}}{{\left(\gamma +\theta \right)}^{a}{\left(1-(\gamma +\theta )\right)}^{2-a}},$$

one can obtain that

$$a=\frac{2}{1+A},$$

where

$$A=\left(\log\frac{\gamma -\theta }{\gamma +\theta }\right)/\left(\log\frac{1-\gamma -\theta }{1-\gamma +\theta }\right).$$

**Figure 2:**
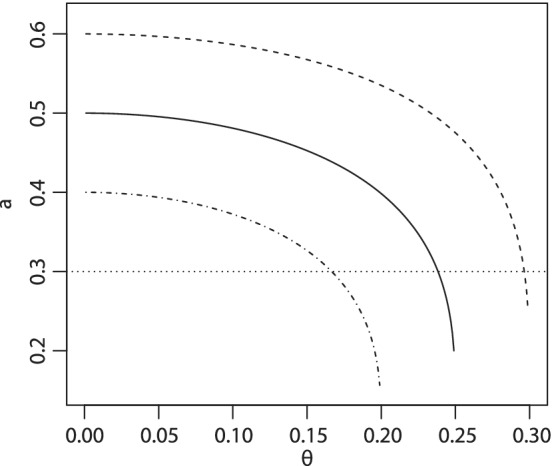
The values of parameter of asymmetry for *γ*=0.20 (dashed‐dotted line), *γ*=0.25 (solid line), *γ*=0.30 (dashed line), and different values of *θ*∈(0,0.35). The horizontal line corresponds to the choice of *a*=0.30 and corresponding half‐width of intervals

Then, for the fixed target value of γ and the half‐width of the interval θ, one can compute the corresponding value of *a*. Figure 2 shows the dependence of the asymmetry parameter on the half‐width θ and the target probabilities γ={0.20,0.25,0.30}.

As θ→0 (the uncertainty issue is prioritized), *a* tends to 2γ, which corresponds to the squared distance allocation rule as shown above. Increasing values of θ correspond to a wider interval in which an investigator prefers a lower toxicity estimate. Consequently, this corresponds to a more conservative allocation and to smaller values of *a*. Note that *a* corresponding to θ≈γ guarantees that for two estimates standing on the same squared distance from the target γ, the dose corresponding to the lower toxicity estimate would be always selected. For example, for the target value γ=0.25 and the half‐width θ=0.245, the corresponding value of *a* is close to 0.3 (marked by the dotted horizontal line in Figure 2).

In the next section, we recall the Bayesian CRM^1^ and incorporate the proposed allocation criterion in the design.

### 2.4 Bayesian CRM with new allocation criterion

Consider a phase I clinical trial with *m* doses and *n* patients. Assume that the DLT probability has the functional form

$$p_{i}=\psi (d_{i},\beta ),$$

where $\beta \in R^{h}$ is a *h*‐dimensional vector of parameters and *d*_*i*_,*i*=1,…,*m*, are standardized dose levels. Denote the prior distribution of β by *f*_0_(.). Assume that *j* patients have already been assigned to doses *d*(1),…,*d*(*j*) and binary responses $Y_{j}={\left[y_{1},\ldots ,y_{j}\right]}^{T}$ were observed. The CRM updates the posterior distribution of β using Bayes theorem

(9) $$f_{j}(\beta )=\frac{f_{j-1}(\beta )\phi (d(j),y_{j},\beta )}{\int_{R^{h}}f_{j-1}(u)\phi (d(j),y_{j},u)\;du}=\frac{f_{0}(\beta )\prod_{i=1}^{j}\phi (d(i),y_{i},\beta )}{\int_{R^{h}}f_{0}(u)\prod_{i=1}^{j}\phi (d(i),y_{i},u)\;du},$$

where

$$\phi (d(j),y_{j},\beta )=\psi {\left(d(j),\beta \right)}^{y_{j}}{\left(1-\psi (d(j),\beta )\right)}^{1-y_{j}}.$$

Then, the posterior mean of the DLT probability for dose *d*_*i*_ after *j* patients is equal to

(10) $${\hat{p}}_{i}^{\left(j\right)}=E(\psi (d_{i},\beta )|Y_{j})=\int_{R^{h}}\psi (d_{i},u)f_{j}(u)\;du.$$

As it was outlined above, the original design uses the following criterion. The dose *d*_*k*_ minimizing

(11) $${\left({\hat{p}}_{i}^{\left(j\right)}-\gamma \right)}^{2},$$

among all *d*_1_,…,*d*_*m*_ is selected for the next group of patients. We propose to replace with the CIBP.

Then, the proposed design can be described as follows.

- Specify the prior distribution *f*_0_, skeleton, the asymmetry parameter *a* and assign the first cohort of patients to the lowest dose.
- After the responses for *j* patients were observed, update the prior distribution, *f*_*j*_, and assign the next cohort of patients to the dose *d*_*k*_ minimizing
  (12) $$E\left(\frac{{\left(\psi (d_{i},\beta )-\gamma \right)}^{2}}{\psi {\left(d_{i},\beta \right)}^{a}{\left(1-\psi (d_{i},\beta )\right)}^{2-a}}\right),$$
  among all *d*_1_,…,*d*_*m*_, where the expectation is found with respect to the posterior probability *f*_*j*_(β).
- Repeated until the maximum number of patients, *n*, has been treated. As the uncertainty and the conservatism is important in the allocation only, the squared distance (11) is used for the final MTD selection.

When implementing and studying the proposed design below, we will concentrate on the one‐parameter power model

(13) $$\psi (d_{i},\beta )=d_{i}^{\exp(\beta )},$$

which was shown to be a powerful tool to identify the MTD.^16^ As there are no concerns about the CRM design to be not aggressive enough, we would concentrate on values *a*≤2γ in the rest of the work.

Finally, it is worth to mention that many implementations of the CRM plug the mean value of β in the model $\psi (d_{i},\hat{\beta })$ instead of using the mean value, $E(\psi (d_{i},\beta )|Y_{j})$. Although no noticeable difference is found in these approaches if a one‐parameter model is used,^17^ it might affect the results significantly if more complex functions are considered.^18^ Therefore, the posterior mean of the new criterion 12 is used. For consistency across all designs, we use the mean probability estimate while performing the original CRM design.

## 3 APPLICATION TO AN ACTUAL CLINICAL TRIAL

### 3.1 Setting

To illustrate the impact of the proposed allocation criterion, we revisit the results of the actual clinical trial of *Everolimus in Patients With HER2‐Overexpressing Metastatic Breast Cancer* (NCT00426556). The study considers three regimens of Everolimus given together with Paclitaxel and Trastuzumab (PT):

1. Daily dosing of Everolimus 5 mg plus PT (*d*_1_).
2. Daily dosing of Everolimus 10 mg plus PT (*d*_2_).
3. Weekly dosing of Everolimus 30 mg plus PT (*d*_3_).

The goal is to find the regimen corresponding to the target toxicity γ=0.3. Note that the amount of the complimentary drugs is fixed during the trial and a clinician is confident in the monotonic relationship of toxicity probabilities for *d*_1_,…,*d*_3_. Thus, the trial can be analyzed using the tools for the single‐agent trials. The aggregated data available by the end of the trial consists of six, 17, and 10 patients being assigned to doses *d*_1_,*d*_2_,*d*_3_, respectively, out of which three, six, and seven experienced DLTs. We revisit the results of this trial using the novel allocation criterion.

We apply the CRM design using the one‐parameter power model 13 by using the robust operational prior distribution β∼𝒩(0,1.34) 5, 19 and the skeleton (0.20,0.30,0.40) with an adequate spacing^20^ and implying that the prior MTD is *d*_2_. We restrict the design so that the dose skipping is not allowed and enforce starting from the lowest dose. Patients are enrolled in cohorts of three. Note that the parameters of the design are the same for both the original CRM and the CRM using the novel allocation rule. The only difference is the criterion for the selection of doses. The original CRM uses the squared distance (11), whereas the CIBP design uses the criterion (12). Following the interpretation of the asymmetry parameter, we fix *a*=0.3 to favor less toxic selections in a wide interval of toxicity probabilities. The designs are implemented using the interactive functions of the bcrm‐package.^21^ We use the aggregated data to generate the responses in one realization of the trial. Clearly, DLTs indicated can appear in any sequence. Therefore, we generate a random sample (without replacement) for each dose to have a specific order of DLTs. We fix this order for both trials. The only exception is that the realization for the first cohort is chosen by us. We consider the influence of this choice later.

### 3.2 Illustration

**Figure 3:**
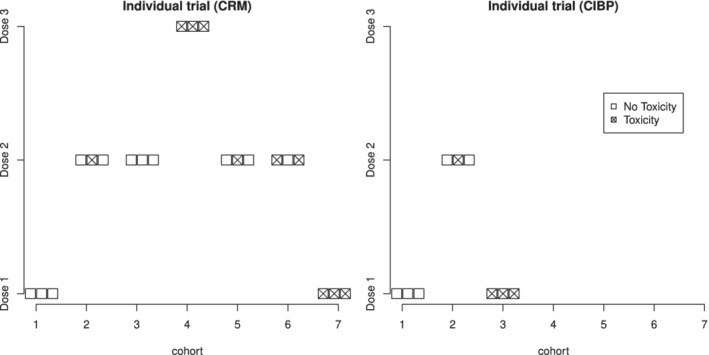
Allocation of seven cohorts in the individual Everolimus trial

The first three patients are assigned to *d*_1_ by construction. We begin by assuming that all three patients have not experienced DLTs. The sequential dose selections for the CRM and CIBP designs, in this case, are given in Figure 3. The values of the criteria after each cohort are given in Table 1.

After no DLTs were observed for the first cohort, the criteria used by CRM and CIBP are both maximized for dose *d*_3_. However, due to the no‐skipping dose restriction, the designs allocate the second cohort of patients to *d*_2_ for which one patient experiences a DLT. Given this toxic outcome, CRM recommends staying at *d*_2_ for the third cohort. By contrast, CIBP recommends returning to the previous dose level due to the conservatism of the criterion. Then, after all patients in cohort 3 (using CIBP) experienced the DLT, the trial would be terminated by a clinician due to safety. At the same time, the trial using the original CRM design proceeds. After no DLTs were observed for cohort 3, *d*_3_ is recommended for cohort 4 in which all patients have DLT. This leads to deescalation to *d*_2_ and after 2 cohorts for which 3 patients (out of 6) had DLT and further de‐escalation to *d*_1_. All 3 patients in cohort 7 experienced DLTs and a clinician terminates a trial due to toxicity. Overall, while the CRM assigned 21 patients and 10 of them experienced DLTs to come to the same conclusion as CIBP, the novel criterion allows reducing the sample size to 9 patients with 4 toxicity outcomes only.

The illustration above demonstrates the allocation if no toxicity outcomes are observed in cohort 1, but other possibilities should be considered as well based on aggregated data. Clearly, the other possibilities are one, two, and three DLTs in the first cohort. Considering these scenarios, it was found that both designs lead to the same allocation of patients and never escalate from dose *d*_1_. It follows that the novel allocation rule leads to the same MTD selection in all possible sequence of outcomes, but results in a fewer or similar number of toxic responses. This motivates a further investigation of the novel criterion in a comprehensive simulation study.

## 4 COMPARISON WITH THE ORIGINAL CRM

### 4.1 Setting

In this section, we compare the traditional one‐parameter CRM to the proposed design in a simulation study. Since the proposed design (Section 2.4) differs from the traditional one‐parameter CRM in the allocation criterion only, any differences in the performances are due to the proposed allocation criterion. We provide a broader comparison with other methods in Section 5.

**Figure 4:**
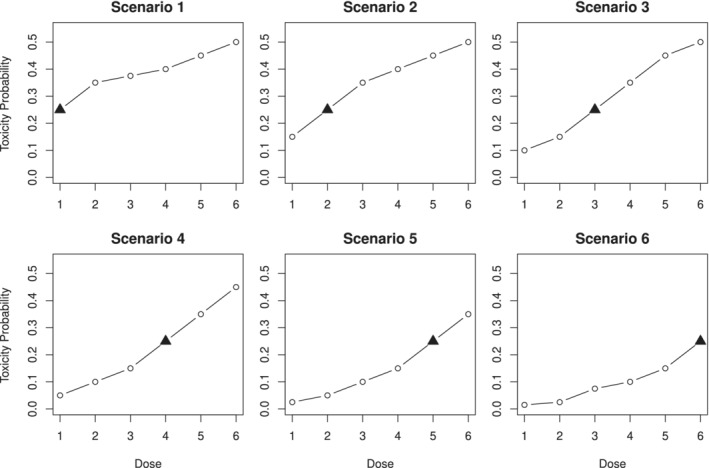
Six considered dose‐toxicity scenarios for the comparison with the CRM. The MTD is marked by the black triangle. CRM, continual reassessment method; MTD, maximum tolerated dose

The single‐agent phase I trial with *m*=6 doses and *n*=30 patients is considered. The goal is to find the MTD corresponding to γ=0.25. We consider six dose‐toxicity scenarios with the target doses located at the dose corresponding to the scenario's number. The shapes of the dose‐toxicity are shown in Figure 4. Toxicity scenarios were chosen “equally difficult” in terms of the optimal nonparametric benchmark.^22^, ^23^ It allows comparing the proportion of correct selections (PCS) between different scenarios. We specify the skeleton for the one‐parameter power model using the package dfcrm and the function getprior using that the prior MTD is *d*_2_ and the half‐width of the equivalent interval is 0.05. The prior distribution of the parameter is chosen to be β∼𝒩(0,1.34).^5^, ^19^, ^24^ Different skeletons corresponding to *d*_3_ and *d*_4_ being the MTD are also investigated and the corresponding (quantitatively similar) results are given in the Appendix. We study the performance of the designs in terms of (i) the PCS, (ii) the accuracy index^7^

$$𝒜=1-m\frac{\sum_{i=1}^{m}{\left(p_{i}-\gamma \right)}^{2}\pi_{i}}{\sum_{i=1}^{m}{\left(p_{i}-\gamma \right)}^{2}},$$

where *p*_*i*_ is the true toxicity probability for *d*_*i*_ and π_*i*_ is the probability to select *d*_*i*_, and (iii) mean number of patients experienced a toxic response (DLT). The first two characteristics measure how accurate a method is in selecting the dose with the desirable toxicity characteristics. Although the PCS focuses solely on the proportion of the MTD selection, the accuracy index also takes into account selections that are not the MTD while treating the error of selecting the dose with the toxicity probability closer to γ=0.25 as less severe. The third characteristic will provide insights into the safety properties of a method. Looking at all of these characteristics together will enable us to choose a design with a desirable accuracy‐toxicity trade‐off. As many different scenarios are considered, one can expect that one design would outperform another in some of them.^25^ Therefore, we focus on average performance: the (geometric) mean accuracy and PCS and the mean number of DLTs across all scenarios.

We consider different values of *a*={0.3,0.4,0.5} corresponding to the approximate half‐width of the intervals θ≈{0.25,0.20,0.00}. The highest value of *a* is chosen to be 2γ, as it is deduced in Section 2.3 that this value corresponds to nearly the same allocation as the CRM and values of *a* below 2γ are expected to result in a more conservative allocation than the CRM—the effect that the proposed design aims to achieve. We denote the CRM with the new escalation criterion using parameter *a* by CIBP(a). The characteristics of all the models compared are evaluated in R 26 using the bcrm‐package.^21^ To accommodate the new criterion, the corresponding modifications to the package were made.

### 4.2 Operating characteristics

Proportions of each dose selections, accuracy index in each scenario, and the average number of patients experienced a DLT for CRM and CIBP designs are given in Table 2. We use 40 000 simulations to declare any difference above 1% as a significant one.

Comparing the performance of CIBP for different values of the asymmetry parameter, one can see that more conservative allocation and selection proportions correspond to CIBP(0.3). The greatest difference can be seen in scenarios 1 and 6. The increase in *a* from 0.3 to 0.5 leads to an increase in the PCS by 5% in the toxic scenario 1 and a decrease in the PCS by 3.5% in the flat scenario 6. This is also reflected in the accuracy indices: CIBP(0.3) corresponds to the highest accuracy index under the toxic scenario 1, whereas the CIBP(0.5) results in the lowest accuracy. In the flat scenario 6, CIBP(0.3) results in the lowest accuracy index with the CIBP(0.5) having the highest accuracy. The differences in the rest of the scenarios are smaller, but still significant. Overall, greater values of *a* favor higher doses to be selected, leading to a higher accuracy index in safe scenarios and a higher average number of toxic responses.

Regarding the comparison of CIBP and CRM, one can find that CIBP(0.4) has a similar PCS and accuracy index, but also a smaller proportion of toxic responses in all considered scenarios. The CIBP(0.5) performs similar (scenarios 2‐5) or better (scenario 6) than CRM at the cost of 1% decrease in the PCS in scenario 1. The most noticeable difference can be observed by comparing CRM with CIBP(0.3). In terms of the PCS and accuracy, CIBP(0.3) outperforms the CRM in the most toxic scenarios 1 and 2, shows comparable performance in scenarios 3 to 5, and results in a lower accuracy index (0.79 against 81 for the CRM) in scenario 6. Interestingly, the accuracy index shades more light on the difference in performance; although the PCS in scenario 6 differs by less than 1%, the CRM tends to allocate more patients to *d*_5_ (difference of 2%). This results in a lower accuracy index for CIBP (0.3) At the same time, CIBP(0.3) outperforms the CRM in terms of the average number of toxic responses in all considered scenario. Although the margin of the difference might be seen to be negligibly small, this improvement results in nearly one fewer patient experiencing a DLT in all scenarios except scenario 1, where the difference is 0.5. Concerning the overall average performances (given in Supporting Information together with the graphical representation of the results), the CIBP with parameters *a*=0.3,0.4,0.5 results in the (geometric) mean of accuracy indices of 0.74, whereas the CRM results in 0.73. At the same time, CIBP designs results in 6.24, 6.68, and 7.12 average DLT response across the scenarios against 7.03 by the CRM. Therefore, CIBP(0.3) and CIBP(0.4) can be considered as a more ethical alternative to the CRM, as it exposes fewer patients to more toxic doses while leading to a slightly greater mean accuracy.

Another valuable feature of the novel allocation criterion is the additional flexibility that allows controlling the number of toxic responses directly. A clinician can choose the parameter *a* based on their conservatism and the range of scenarios of interest. For instance, a clinician might be ready to sacrifice the PCS in the flat scenario 6 for the sake of not selecting overly toxic dose in scenario 1. The new criterion enables such an option. At the same time, the design preserves its simplicity and does not result in any extra computational costs.

## 5 COMPARISON WITH ALTERNATIVE APPROACHES

### 5.1 Setting

Alternative criteria for solving the ethical and uncertainty issues using the two‐parameter logistic model

$$\psi (d_{i},\beta_{1},\beta_{2})=\frac{\exp(\beta_{1}+\beta_{2}d_{i})}{1+\exp(\beta_{1}+\beta_{2}d_{i})},$$

were proposed^4^ using the EWOC design. However, as stated above, the EWOC can result in a systematic underestimation of the MTD. Therefore, some modifications were proposed.^6^, ^7^ The main idea beyond the modifications is to use a changing parameter α_*n*_ in the criterion (3) rather than a fixed value of α. The detailed description of these modifications can be found in Reference 7. Alternatively, the BLRM method^8^ also using the two‐parameter logistic model and a loss function can be used. In this section, we compare the performance of the original one‐parameter CRM and the proposed approach using the novel allocation rule to these designs.

**Figure 5:**
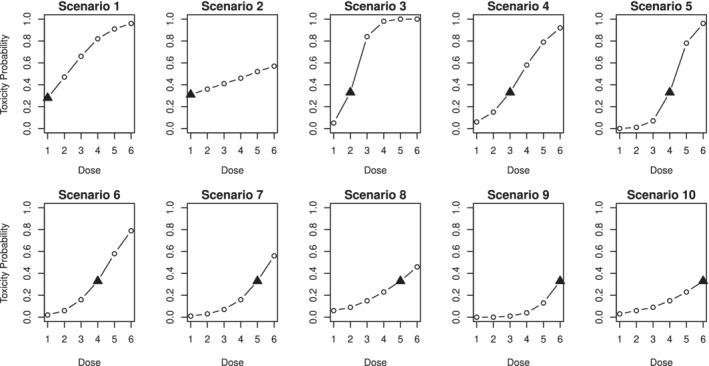
Ten considered dose‐toxicity scenarios for the comparison with EWOC. The MTD is marked by the black triangle. EWOC, escalation with overdose control; MTD, maximum tolerated dose

We consider the setting by Wheeler et al^7^ for discrete dose levels. There are *n*=40 patients and *m*=6 doses in the trial. The goal is to find the MTD corresponding to the γ=0.33. The original scenarios are given in Figure 5.

For the CRM and CIBP designs, the prior distribution of β is specified as in the previous section. The only difference is the skeleton, which is now set using the same information as by Wheeler et al^7^: the prior MTD is *d*_3_. Assuming that ethical issues are of the greater interest in this trial, we consider several values of *a*<2γ=0.66 that would result in a more conservative allocation of patients than the CRM, namely, *a*={0.65,0.5,0.25,0.10}.

We compare the performance of the proposed approach to

- EWOC design—the original EWOC design using fixed α=0.25.
- TR design^6^ (where TR stands for the authors of the proposed approach, Tighiouart and Rogatko) using α_2_=…=α_9_=0.25, $\alpha_{n}=\min(\alpha_{n-1}+0.05,0.50)$ for all future patients.
- Toxicity‐dependent feasibility bound design (TDFB)^7^ using
  $$\alpha_{n+1}=\min(0.50,\alpha_{min}+(0.50-\alpha_{min}\frac{n-1-\sum_{i=1}^{n}y_{i}}{S}),$$
  where $n-1-\sum_{i=1}^{n}y_{i}$ is the number of patients with no DLTS, α_min_=0.25 and $S=12\frac{2}{3}$. For both modifications of the EWOC design above, we use the parameters as in Reference 7.
- BLRM design^8^ which uses the loss function for the decision. Following the original proposal, we use the same bivariate normal prior distribution for parameters as in the original work and adapt the toxicity intervals for the loss function for γ=0.33
  $$L=\left\{\begin{array}{ll} 1 & \text{if}\;p\in (0.00,0.26)\\ 0 & \text{if}\;p\in (0.26,0.41)\\ 1 & \text{if}\;p\in (0.41,0.66)\\ 2 & \text{if}\;p\in (0.66,1.00) \end{array}\right..$$

In addition, we investigate a modification of the one‐parameter CRM model that is used in practice but, to our best knowledge, was not yet extensively studied and compared in a simulation study.

- This modification of the CRM uses the squared distance criterion but allows for the allocation to the doses with the estimated toxicity strictly below the target toxicity γ. We will denote this model as “CRM (M)” where M stands for the modified.

As in the previous section, we are primarily interested in the accuracy‐toxicity trade‐off for each method. Therefore, we study the performance of the designs in terms of (i) the PCS, (ii) the accuracy index, and (iii) the average number of DLTs experienced by the patients in one trial. Again, we will focus on the average performances across the scenarios.

### 5.2 Operating characteristics

**Figure 6:**
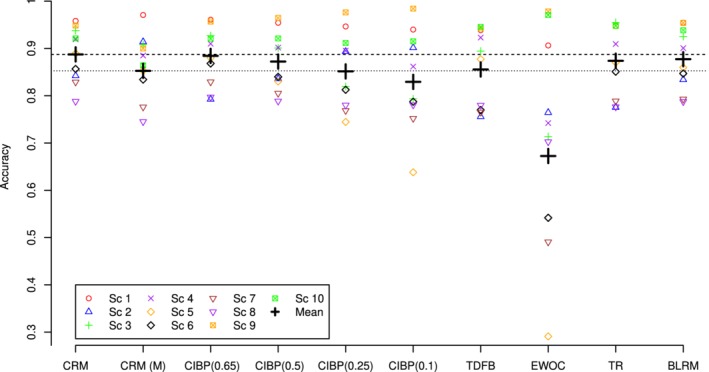
Accuracy indices and mean accuracy indices for CRM, CRM(M), CIBP using *a*={0.65,0.5,0.25,0.10}, TDFB, EWOC, TR, and BLRM designs. The dashed horizontal line corresponds to the accuracy of the CRM, and the dotted line corresponds to the accuracy of the CRM(M). Results are based on 2000 simulations. BLRM, Bayesian logistic regression model; CIBP, convex infinite bounds penalization; CRM, continual reassessment method; EWOC, escalation with overdose control; PCS, probability of correct selections; TDFB, toxicity‐dependent feasibility bound design [Color figure can be viewed at http://wileyonlinelibrary.com]

**Figure 7:**
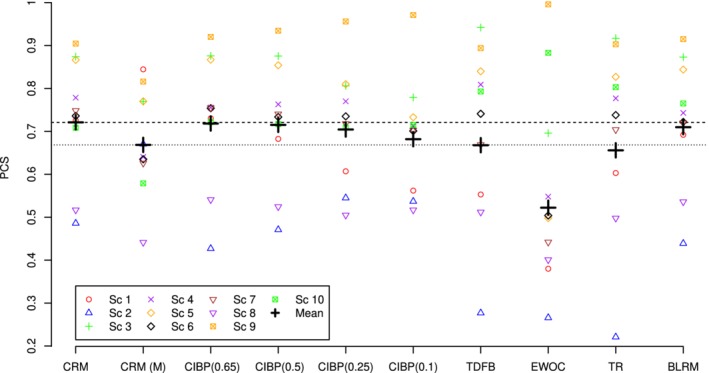
PCS and the corresponding means for CRM, CRM(M), CIBP using *a*={0.65,0.5,0.25,0.10}, TDFB, EWOC, TR, and BLRM designs. The dashed horizontal line corresponds to the PCS of the CRM, and the dotted line corresponds to the PCS of the CRM(M). Results are based on 2000 simulations. BLRM, Bayesian logistic regression model; CIBP, convex infinite bounds penalization; CRM, continual reassessment method; EWOC, escalation with overdose control; PCS, probability of correct selections; TDFB, toxicity‐dependent feasibility bound design [Color figure can be viewed at http://wileyonlinelibrary.com]

**Figure 8:**
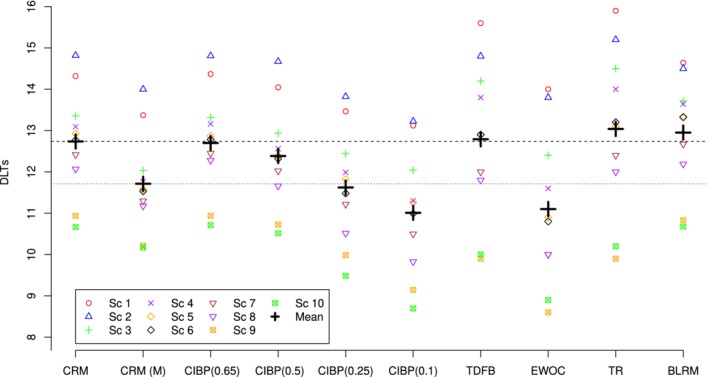
DLTs and mean number of DLTs for CRM, CRM(M), CIBP using *a*={0.65,0.5,0.25,0.10}, TDFB, EWOC, TR, and BLRM designs. The dashed horizontal line corresponds to the mean number of DLTs of the CRM and the dotted line corresponds to the CRM(M). Results are based on 2000 simulations. BLRM, Bayesian logistic regression model; CIBP, convex infinite bounds penalization; CRM, continual reassessment method; DLTs, dose‐limiting toxicities; EWOC, escalation with overdose control; PCS, probability of correct selections; TDFB, toxicity‐dependent feasibility bound design [Color figure can be viewed at http://wileyonlinelibrary.com] [Correction added on 24 January 2020 after first online publication: Figure 8 has been corrected.]

The accuracy indicies and the PCS for the CRM, CRM (M), CIBP using *a*={0.65,0.5,0.25,0.10}, TDFB, EWOC, TR, and BLRM design in the individual scenarios and corresponding means are given in Figures 6 and 7, respectively. As mentioned above, the accuracy indices and PCS themselves are of a limited interest when considered alone without taking the safety into account. To reflect on the accuracy‐toxicity trade‐off, the mean number of DLTs in all considered designs is shown in Figure 8.

Comparing CIBP for different values of *a*, one can see that both mean accuracy index and the PCS decrease as parameter *a* increases. Due to a more conservative allocation for smaller values of *a*, fewer patients are assigned to doses in the neighborhood of the MTD. The decrease in the accuracy index and PCS is, however, rather small—from 0.88 and 0.72 using *a*=0.65 to 0.83 and 0.68 using *a*=0.1, respectively. The most noticeable drop across scenarios can be found in scenario 5—0.20. The variance of the accuracy indexes increases with decreasing *a*—the more conservative designs lead to a better performance in toxic scenarios 1 to 3 for the cost of a less accurate performance in flat scenarios 8 to 10. Regarding the safety of the CIBP designs, lower values of *a* result in fewer DLTs on average across all scenarios. As a result, the mean number of DLTs is decreased by approximately two toxic responses comparing CIBP(0.65) and CIBP(0.10).

Comparing different approaches, we will start by presenting the results in the groups of the methods that result in similar characteristics in terms of one of the accuracy measures (accuracy index or PCS) and highlight the difference in other measures. This will allow to focus on the accuracy‐toxicity trade‐off of the methods. First, CRM and CIBP(0.65) correspond to the highest mean accuracy index and mean PCS and the same mean number of DLTs across the scenarios. Therefore, these two designs perform comparably in terms of the accuracy‐toxicity trade‐off. At the same time, CIBP being a more flexible design allows to tackle the balance between accuracy and safety. Specifically, by decreasing the asymmetry parameter to *a*=0.5, one can decrease the mean number of DTLs by nearly 0.35 for the price of 1.5% decrease in the accuracy index and 0.6% decrease in the mean PCS.

The TR, BLRM, and CIBP(0.5) result in a comparable but slightly lower mean accuracy index than the CRM. The TR design results in nearly 5% lower mean PCS than BLRM and CIBP(0.5). At the same time, CIBP(0.5) results in a lower mean number of DLTs across scenarios than both of these methods: 12.4 against nearly 13 for the TR and BLRM. Consequently, CIBP(0.5) can improve the safety of the TR and BLRM while not compromising (or even improving) the accuracy of selection.

The CRM(M), TDFB, and CIBP(0.25) perform similarly in terms of the mean accuracy index, but CIBP(0.25) results in a slightly higher (by 3%) PCS than the corresponding comparators. In terms of the safety of these methods, CIBP(0.25) and CRM(M) results in the lowest mean number of DLTs with the difference of nearly 1 more toxic response compared with the TDFB. This means that CIBP(0.25) has nearly the same safety properties as CRM(M) but can improve accuracy while improving both of these factors over TDFB.

Finally, we compare two of the remaining designs, EWOC and CIBP(0.1). Both of these designs result in the lowest (and similar) average number of DLTs across the scenarios. At the same time, the EWOC results in the least mean accuracy index due to the large MTD underestimation in scenarios 5 to 7. The mean accuracy index and the PCS associated with the most conservative CIBP(0.10) are greater than those associated with EWOC by 0.15 and 0.16, respectively. Consequently, the proposed design with parameter *a*=0.1 retains the safety properties of the EWOC but results in a more accurate MTD selection.

Overall, it is found that the original CRM results in the highest accuracy index comparable with a number of other approaches. At the same time, it results in a high average number of toxic responses, but its original version does not allow to incorporate the conservatism of an investigator in the allocation rule. By restricting the allocation rule of the original CRM to select doses that are strictly below γ can improve the safety of the method (for the price in the accuracy), but a better accuracy‐toxicity balance could be achieved using the CIBP design. Generally, the proposed CIBP criterion allows tuning the trade‐off between accuracy and safety explicitly. At the same time, in contrast to TDFB and TR, the proposed design does not change the parameter of conservatism as more patients are trialed. It requires only one extra parameter to be specified. However, one can find a value of parameter *a* that would lead to a better trade‐off between the accuracy of the MTD selection and the safety, specifically, by improving one aspect while not compromising another.

## 6 DISCUSSION

A novel dose‐escalation criterion for the allocation of patients is introduced in this work. The criterion requires only one additional parameter, the parameter of asymmetry, which has clear intuitive interpretation and can be easily tuned according to the purposes of the investigator to achieve a desirable trade‐off between accuracy and the number of patients experiencing DLTs. It is found that incorporated into the one‐parameter power Bayesian CRM design, the new criterion, for particular values of the asymmetry parameter, results in nearly the same performance as the original CRM with the squared distance criterion (11) in the terms of the accuracy and safety, but for lower values of the parameter can achieve better accuracy‐toxicity trade‐offs than a number of dose‐finding designs currently used in practice. Specifically, compared with the EWOC, its different variations and two‐parameter BLRM design, it is found that the CIBP design for various values of the parameter leads to either (i) nearly the same average accuracy index and/or PCS but fewer average number of DLTs (comparing CIBP(0.5) with TDFB, TR, and BLRM), or (ii) higher average accuracy and/or PCS but the same number of DLTs (comparing CIBP(0.1) with EWOC). Concerning the modified CRM criterion using the truncated squared distance, the proposed criterion is found to result in the same accuracy index, higher average PCS, and slightly fewer number of DLTs. This makes the proposed design a more ethically viable alternative over some of the existing methods considered in this work. Finally, the asymmetry parameter allows balancing of the characteristics within a unified framework, so the desirable accuracy‐toxicity trade‐off can be achieved within one dose‐escalation method rather than using different methods with each requiring independent calibration.

One of the crucial parts of the proposed criterion is the asymmetry parameter. In the actual trial, this parameter can be chosen together with clinicians using the parameter's interpretation (Section 2.3) and considering the results of the simulation study (in clinically feasible scenarios). Moreover, a recently published tool to calibrate the parameters of the CRM, namely, dose transition pathways (DTP)^27^ can be used. This tool can also accommodate the inclusion of the proposed parameter: the clinicians are provided with the decision trees obtained by DTP for various values of the parameter *a* and the parameter is calibrated until the escalation/deescalation rules are consistent with clinicians' knowledge.

Although we have considered the form of the allocation criterion with the squared distance being in the numerator, as pointed one by one of the reviewer, one could also consider a similar form but using the absolute distance,

(14) $$\delta_{1}(p,\gamma )=\frac{\left|p-\gamma \right|}{p^{a}{\left(1-p\right)}^{2-a}}.$$

In small‐scale settings, the absolute distance will penalize more than the squared distance. This implies that for the same values of *a* one can expect that the criterion (14) would favor the accuracy of the design rather than safety. Nevertheless, it was found that the proposed criterion and the criterion (14) result in the same recommendation in most situations. Simulations in the setting of Section 5 are given in the Supporting Information.

It is important to reiterate that we focused on the application of the novel criterion to the one‐parameter power model throughout this work, as it has been shown^5^, ^17^, ^19^, ^28^ to be able to identify the MTD with a high probability. Note, however, that the proposed criterion is generic and can be applied to any parametric model (for instance, the two‐parameter logistic model) if it is preferred by an investigator. Moreover, the application of the criterion was demonstrated in the context of a single‐agent trial only. As there are generalizations of the CRM design for more complex studies, it is also of interest to consider the application of the novel allocation rule to dose‐combination and dose‐schedule trials including the case of delayed toxicity responses.

## Supporting information



Data S1 Supporting Information
