# Supplementary material for: Improving safety of the continual reassessment method via a modified allocation rule
Source: Stat Med. 2019 Dec 20;39(7):906–22. doi: 10.1002/sim.8450 (PMC7064916; doi:10.1002/sim.8450)
Supplement: Supplementary file 1 — Data S1 Supporting Information [file SIM-39-906-s001.pdf]

Received: Added at production | Revised: Added at production | Accepted: Added at production
DOI: xxx/xxxx

ARTICLE TYPE

Improving Safety of the Continual Reassessment Method via a Modified Allocation Rule. Supplementary Materials.

Pavel Mozgunov* | Thomas Jaki

[1]Department of Mathematics and Statistics, Lancaster University, Lancaster, UK

Correspondence
*Corresponding author Email: p.mozgunov@lancaster.ac.uk

Present Address
B floor, Fylde College, Lancaster University, Bailrigg, Lancaster, LA1 4YF, UK

FIGURES OF THE ACCURACY INDEX, PCS AND AVERAGE DLTS FOR METHODS IN SECTION 4

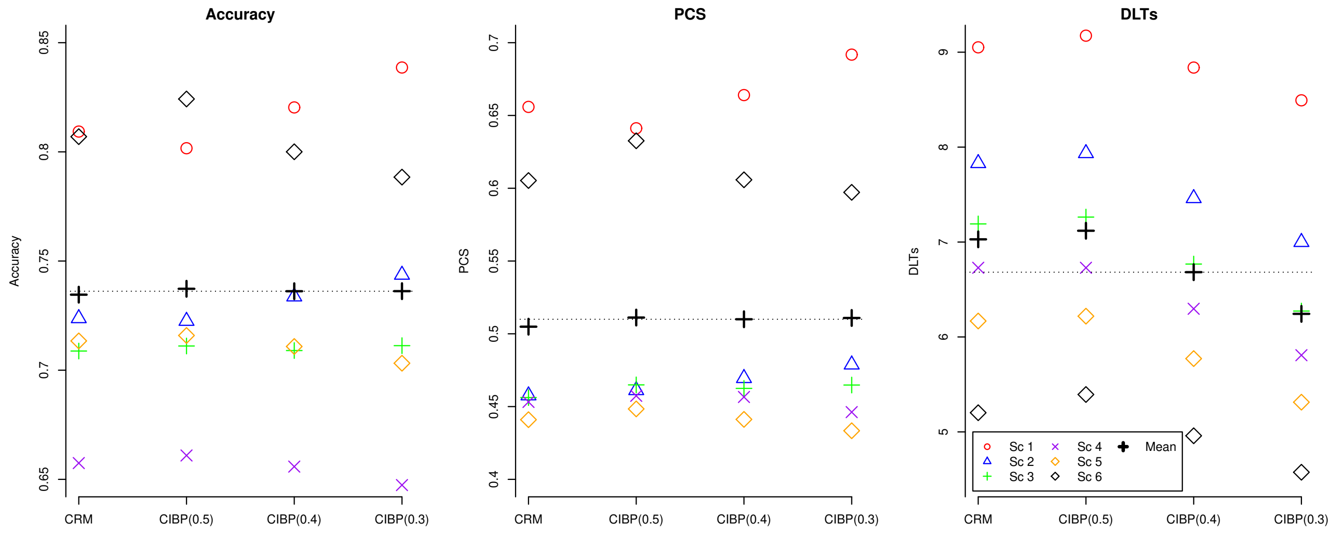

FIGURE 1 Accuracy indices, PCS and average number of DLTs and corresponding means for CRM, CIBP using $a = \{0.5, 0.4, 0.3\}$ designs. Results are based on 40000 simulations.

RESULTS FOR DIFFERENT PRIOR MTD IN SECTION 4

TABLE 1 Proportions of doses selections, accuracy index (Accuracy) and average number of DLTs (DLTs) in one trial for the CRM and the CIBP using $a = \{0.3, 0.4, 0.5\}$ and the prior parameter as described in Section 4, but the prior MTD d_3 is used for the skeleton construction. Results are based on 40000 simulations.

	d_1	d_2	d_3	d_4	d_5	d_6	Accuracy	DLTs
				Scenario 1				
Toxicity	**25.00**	35.00	37.50	40.00	45.00	50.00		
CIBP (0.3)	67.59	22.50	6.44	2.60	0.74	0.13	0.83	8.62
CIBP (0.4)	64.12	22.53	8.29	3.77	1.08	0.21	0.80	8.99
CIBP (0.5)	61.78	22.23	9.61	4.80	1.30	0.28	0.78	9.39
CRM	63.27	21.28	9.45	4.52	1.28	0.21	0.79	9.31
				Scenario 2				
Toxicity	15.00	**25.00**	35.00	40.00	45.00	50.00		
CIBP (0.3)	22.86	47.00	23.37	5.45	1.15	0.17	0.73	7.18
CIBP (0.4)	22.89	45.59	23.64	6.25	1.40	0.23	0.72	7.69
CIBP (0.5)	23.47	44.65	23.12	6.97	1.49	0.29	0.71	8.14
CRM	24.38	44.26	22.64	6.81	1.64	0.27	0.71	8.17
				Scenario 3				
Toxicity	10.00	15.00	**25.00**	35.00	45.00	50.00		
CIBP (0.3)	3.76	23.83	46.95	21.62	3.51	0.33	0.71	6.52
CIBP (0.4)	3.84	23.95	46.45	21.98	3.47	0.32	0.71	6.98
CIBP (0.5)	3.53	23.99	46.36	22.17	3.56	0.40	0.71	7.53
CRM	3.52	24.57	46.51	21.54	3.50	0.37	0.71	7.55
				Scenario 4				
Toxicity	5.00	10.00	15.00	**25.00**	35.00	45.00		
CIBP (0.3)	0.17	4.45	25.60	46.06	20.42	3.29	0.66	6.03
CIBP (0.4)	0.16	4.15	25.51	46.38	20.60	3.20	0.66	6.49
CIBP (0.5)	0.15	4.00	25.66	46.66	20.38	3.14	0.67	7.01
CRM	0.18	4.08	26.25	46.39	20.24	2.85	0.67	7.02
				Scenario 5				
Toxicity	2.50	5.00	10.00	15.00	**25.00**	35.00		
CIBP (0.3)	0.00	0.27	5.71	27.24	44.13	22.64	0.71	5.52
CIBP (0.4)	0.00	0.24	5.06	26.41	45.88	22.41	0.72	5.99
CIBP (0.5)	0.00	0.27	4.97	27.23	45.48	22.05	0.72	6.42
CRM	0.00	0.25	4.58	27.98	45.88	21.31	0.73	6.47
				Scenario 6				
Toxicity	1.50	2.50	10.00	10.00	15.00	**25.00**		
CIBP (0.3)	0.00	0.02	2.42	9.37	26.83	61.36	0.80	4.75
CIBP (0.4)	0.00	0.02	1.74	7.62	27.18	63.44	0.82	5.16
CIBP (0.5)	0.00	0.05	1.74	7.75	27.89	62.58	0.82	5.46
CRM	0.00	0.04	1.29	6.63	28.55	63.49	0.83	5.51

TABLE 2 Proportions of doses selections accuracy index (Accuracy) and average number of DLTs (DLTs) in one trial for the CRM and the CIBP using $a = \{0.3, 0.4, 0.5\}$ and the prior parameter as described in Section 2, but the prior MTD d_4 is used for the skeleton construction. Results are based on 40000 simulations.

	d_1	d_2	d_3	d_4	d_5	d_6	Accuracy	DLTs
			Scenario 1					
Toxicity	**25.00**	35.00	37.50	40.00	45.00	50.00		
CIBP (0.3)	64.38	23.48	7.74	3.21	1.03	0.15	0.81	8.77
CIBP (0.4)	62.80	22.43	9.04	4.15	1.35	0.24	0.79	9.17
CIBP (0.5)	59.16	22.61	10.51	5.68	1.73	0.31	0.76	9.58
CRM	60.09	21.50	10.73	5.63	1.74	0.30	0.76	9.59
			Scenario 2					
Toxicity	15.00	**25.00**	35.00	40.00	45.00	50.00		
CIBP (0.3)	21.23	46.23	24.93	6.07	1.36	0.18	0.73	7.38
CIBP (0.4)	21.78	44.21	25.04	6.94	1.74	0.29	0.71	7.94
CIBP (0.5)	22.46	43.10	24.44	7.82	1.87	0.31	0.69	8.37
CRM	23.79	42.60	23.60	7.78	1.92	0.32	0.69	8.45
			Scenario 3					
Toxicity	10.00	15.00	**25.00**	35.00	45.00	50.00		
CIBP (0.3)	3.50	22.07	47.11	23.09	3.92	0.31	0.71	6.71
CIBP (0.4)	3.37	21.87	46.68	23.73	3.97	0.38	0.70	7.29
CIBP (0.5)	3.54	22.51	46.14	23.47	3.93	0.41	0.70	7.70
CRM	3.53	23.31	45.96	22.88	3.89	0.44	0.70	7.86
			Scenario 4					
Toxicity	5.00	10.00	15.00	**25.00**	35.00	45.00		
CIBP (0.3)	0.13	3.62	23.72	47.04	22.04	3.44	0.67	6.26
CIBP (0.4)	0.16	3.71	23.79	47.30	21.75	3.29	0.67	6.77
CIBP (0.5)	0.15	3.67	24.02	47.06	21.78	3.31	0.67	7.20
CRM	0.15	3.56	24.48	47.12	21.39	3.30	0.67	7.37
			Scenario 5					
Toxicity	2.50	5.00	10.00	15.00	**25.00**	35.00		
CIBP (0.3)	0.00	0.20	4.69	25.61	45.86	23.64	0.73	5.73
CIBP (0.4)	0.00	0.22	4.35	25.54	46.44	23.44	0.73	6.25
CIBP (0.5)	0.00	0.21	4.14	25.68	46.36	23.61	0.73	6.63
CRM	0.00	0.23	4.09	26.50	46.24	22.94	0.73	6.74
			Scenario 6					
Toxicity	1.50	2.50	10.00	10.00	15.00	**25.00**		
CIBP (0.3)	0.00	0.03	1.74	8.63	26.63	62.97	0.82	4.90
CIBP (0.4)	0.00	0.03	1.33	6.53	25.96	66.16	0.84	5.40
CIBP (0.5)	0.00	0.02	1.24	6.20	26.59	65.94	0.84	5.66
CRM	0.00	0.05	1.17	6.44	27.95	64.38	0.84	5.66

ABSOLUTE DISTANCE MODIFICATION OF THE CRITERION

While the main focus of the manuscript is to study the behaviour of

$$\delta(p, \gamma) = \frac{(p-\gamma)^2}{p^a(1-p)^{2-a}}, \tag{1}$$

as rightfully noted by one of the reviewers, one can also consider some variants of the proposal. For example, one can replace the squared distance in numerator by the absolute distance,

$$\delta_1(p, \gamma) = \frac{|p-\gamma|}{p^a(1-p)^{2-a}} \tag{2}$$

as, in the small scale setting, the absolute distance would penalize more than the squared distance.

First of all, it is of interest to investigate in what situations the two criteria lead to different dose recommendations. To demonstrate this, we consider pairs of probabilities of toxicity (where in each pair the first value (x-axis in Figure 2) corresponds to the toxicity probability at dose 1, and the second value (y-axis in Figure 2) to the toxicity probability at dose 2) on the grid (with step 0.01) and find for which pairs the measures are in agreement and for which they are not. The results are given in Figure 2

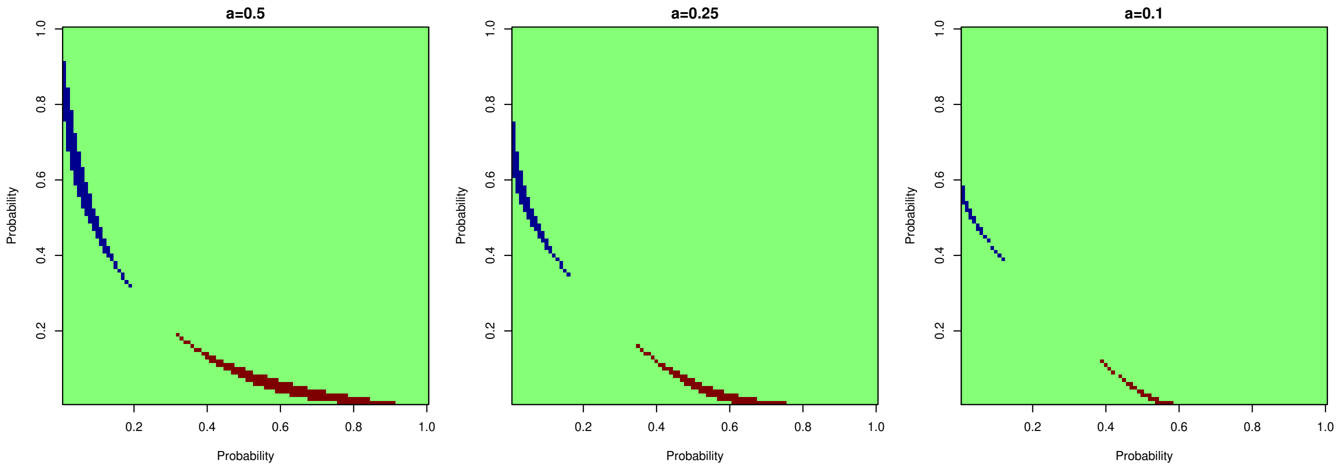

FIGURE 2 The pairs of the probabilities (i) when the proposed criterion using the squared and absolute distances are in agreement (green), (ii) when the probability on the x-axis is favoured over the probability on the y-axis by the absolute distance criterion but not the squared distance criterion (red), (iii) when the probability on the x-axis is favoured over the probability on the y-axis by the squared distance criterion but not the absolute distance criterion (blue).

Figure 2 shows the pairs of the probabilities when (i) both criteria are in agreement (green), (ii) when the probability on the x-axis is favoured (corresponds to the lower values of the criterion) over the probability on the y-axis by the absolute distance criterion but not the squared distance criterion (red), (iii) when the probability on the x-axis is favoured over the probability on the y-axis by the squared distance criterion but not the absolute distance criterion (blue). Indeed, one can note that that the absolute distance criterion tends to favour greater values of p for some pairs implying a more aggressive design. At the same time, it is apparent that the proportion of pairs when the criteria are not in agreement is small and continue to decreases as a decreases.

To investigate the practical differences, we have studied the characteristics of this allocation criterion incorporated into the one-parameter CRM model with the rest of the parameters being as specified in the main body of the manuscripts in the simulation scenarios described in Section 5. Below, we present the corresponding results and compare the performance to the originally proposed measure for the same values of the parameter a.

For $a = 0.5$, differences in all characteristics are marginal with slightly higher values for the absolute distance criterion. As the numerator now penalises the distance between p and γ more severely, this results in a more aggressive allocation but also higher

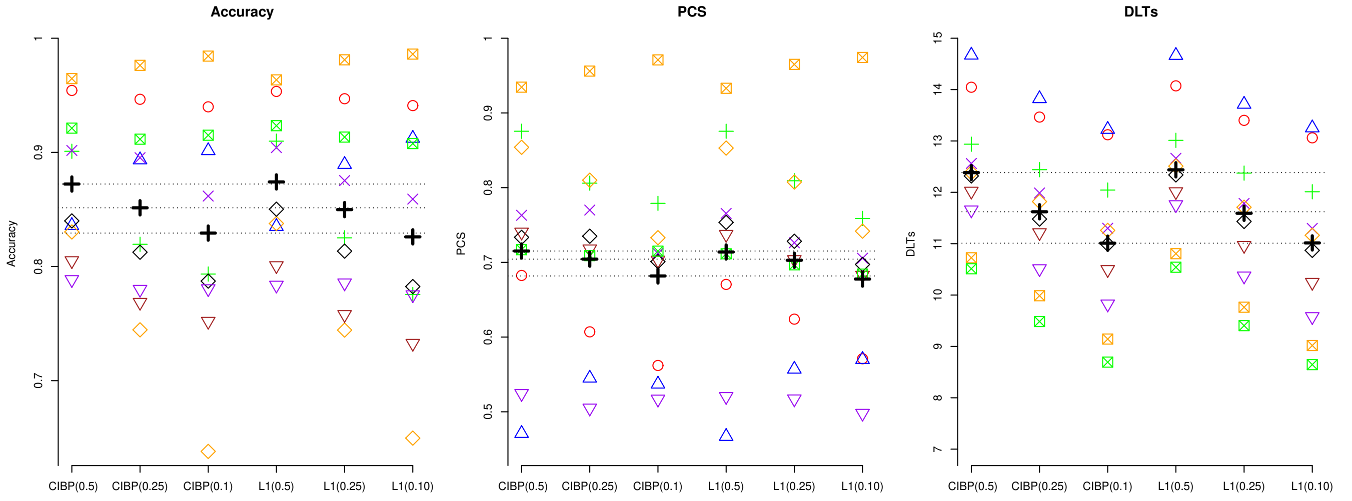

FIGURE 3 Accuracy, PCS, average number of DLTs by the CIBP design and the one-parameter CRM design with the criterion (2) (denoted by L1) using the same value of the parameter $a = 0.5, 0.25, 0.10$ in 10 scenarios considered in Section 5 of the manuscript. Results are based on 2000 simulations.

accuracy for the same values of parameters a. As a decreases, the difference gets even smaller. Therefore, one can conclude that these two measures perform similarly in the considered scenarios.

□
